# Spatial congruency bias in identifying objects is triggered by retinal position congruence: Examination using the Ternus-Pikler illusion

**DOI:** 10.1038/s41598-020-61698-5

**Published:** 2020-03-13

**Authors:** Kyoshiro Sasaki, Atsunori Ariga, Katsumi Watanabe

**Affiliations:** 1 Waseda University, Tokyo, Japan; 2 Kyushu University, Fukuoka, Japan; 3 Hiroshima University, Hiroshima, Japan; 4 University of New South Wales, Sydney, Australia; 5 Japan Society for the Promotion of Science, Tokyo, Japan

**Keywords:** Object vision, Human behaviour

## Abstract

When two different objects are sequentially presented at the same location, the viewer tends to misjudge them as identical (spatial congruency bias). The present study examined whether the spatial congruency bias would involve not only retinotopic but also non-retinotopic processing using the Ternus-Pikler illusion. In the experiments, two objects (central and peripheral) appeared in an initial frame. The target object was presented in the central area of the display, while the peripheral object was either on the left or right side of the target object. In the second frame, the target object was again presented in the central area, and the peripheral object was on the opposite side. Two kinds of inter-stimulus intervals were used. In the no-blank condition, the target object was perceived as stationary, and the peripheral object appeared to move to the opposite side. However, in the long-blank condition, the two objects were perceived to move together. Participants judged whether the target objects in the two frames were identical. As a result, the spatial congruency bias occurred irrespective of the ISI conditions. Our findings suggest that the spatial congruency bias is mainly based on retinotopic processing.

## Introduction

To smoothly interact with objects, one’s visual system must adequately encode and distinguish them in the temporal domain. Fundamentally, various features and properties (e.g., shape, colour, location) of each object are parallelly processed in different areas of the brain, after which they are integrated. This idea is known as the *binding problem*^1–7^. Object location, in particular, seems to be special in the binding problem. Classical and dominant theories posit that object location serves as a pointer, index, or object file to assign various kinds of features to an object^8,9^. Thus, the object’s location is highly important in establishing and maintaining the object representation not only in a spatial domain but also in a temporal domain.

A recent study has reported an interesting phenomenon reflecting this inseparable link between a location and identity of object: *spatial congruency bias*^10^. Spatial congruency bias is the phenomenon in which, when two different objects are sequentially presented at the same location, their identities tend to be misjudged as identical. This bias is found in various kinds of judgments regarding object identity: shape figure, colour, orientation, and human faces^10–12^. The spatial congruency bias is assumed to occur because location plays roles as pointer, index, or object file to bind object features^8,9^ and thus location is used as valid cues for object identification, which then biases judgments of their identity^10–12^. However, it is unclear which locational information, retinotopic locational information or non-retinotopic locational information, is responsible for the spatial congruency bias. A previous study used the Saccadic Stimulus Presentation Paradigm (SSPP) and found that the spatial congruency bias occurred only based on the congruency of retinotopic coordinates^12^. That said, it was also pointed out that when using SSPP, the eye motor system made interpretation of the findings complicated^13^. Thus, it is deemed valuable to simply examine whether the spatial congruency bias is based on retinotopic processing in the absence of eye movements; the present study addressed this issue.

**Figure 1:**
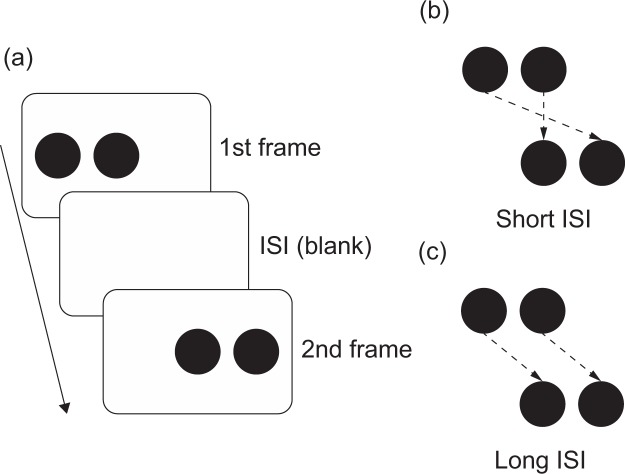
A schematic representation of the Ternus-Pikler illusion. (**a**) shows the method of the presentation of stimuli. (**b,c**) show examples of element and group motions, respectively. The dotted line indicates the perceived motion of the elements.

The Ternus-Pikler paradigm is an effective method used to examine whether phenomena are based on non-retinotopic processing in the absence of eye movements^13–20^. The Ternus-Pikler illusion is a visual illusion of apparent motion^21^ and consists of two frames separated by a short or long blank (inter-stimulus interval [ISI]; Fig. 1a). Both frames contain two elements (e.g., circles in Fig. 1) and the difference between the frames is the position of the elements: the elements shifted horizontally between the frames. In this case, two kinds of apparent motion are perceived depending on the length of the ISI. For the short ISI (<30 ms), the element on the side is perceived to move toward the other side, while the centre element is perceived to be stationary (Element motion; Fig. 1b). Element motion stems from motion correspondence based on retinotopic information. In contrast, for the long ISI (>50 ms), the two elements are perceptually grouped and perceived to shift together (Group motion; Fig. 1c); group motion stems from motion correspondences based on non-retinotopic information. Taken together, the central element is perceived to be stationary in the short ISI, while it is seen to move horizontally in the long ISI.

**Figure 2:**
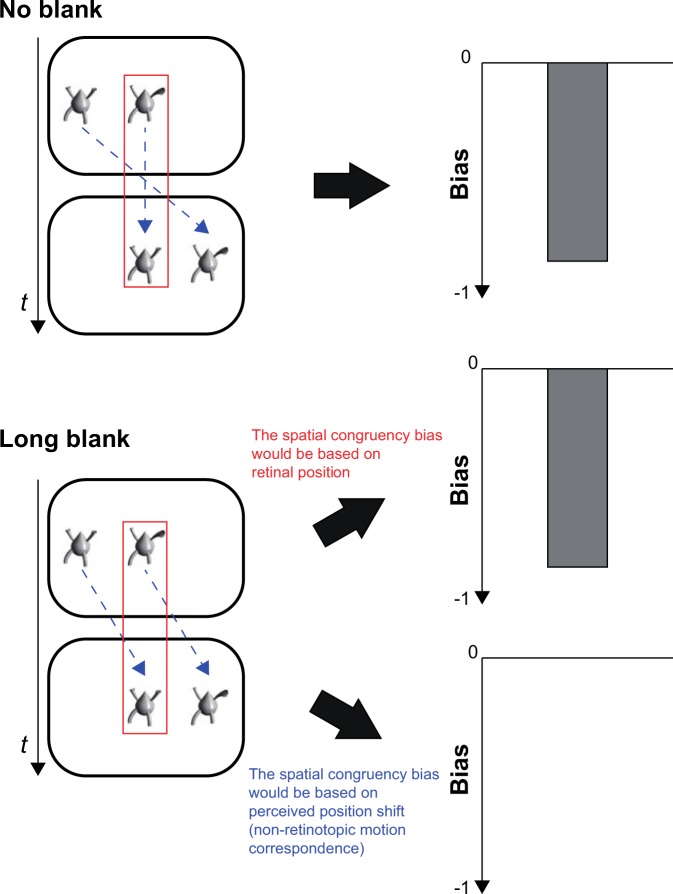
The predictions of the present study. The blue dotted arrows indicate the perceived motion of the objects. The red frames indicate the retinal location of the targets.

The present study aimed to examine whether the spatial congruency bias would involve retinotopic rather than non-retinotopic processing. In two experiments, we asked participants to judge whether the central object (target) was identical between the first and second frames. When no blank existed between the frames, the central objects were perceived to be stationary because of retinotopic motion correspondence. Thus, we predicted that the spatial congruency bias would occur in this case because both the retinal and perceived locations of the targets were constant between the frames (Fig. 2). On the other hand, when a long blank existed between the frames, the central objects were perceived to move horizontally because of non-retinotopic motion correspondence; however, the retinal location of the targets was constant between the frames. In this case, two predictions were possible. If the spatial congruency bias was based on retinotopic processing, the bias (i.e., the tendency to judge two different objects at the same location as identical) would occur in the long-blank condition owing to congruency of the retinal location of the targets even though they were spatiotopically different. Meanwhile, if the spatial congruency bias stemmed from non-retinotopic processing, the retinal-location-based bias would not be observed for the targets that were spatiotopically different between the frames in the 200-ms condition even though they were retinotopically same.

## Results

### Experiment 1: Judgment of the identity of the central object

**Figure 3:**
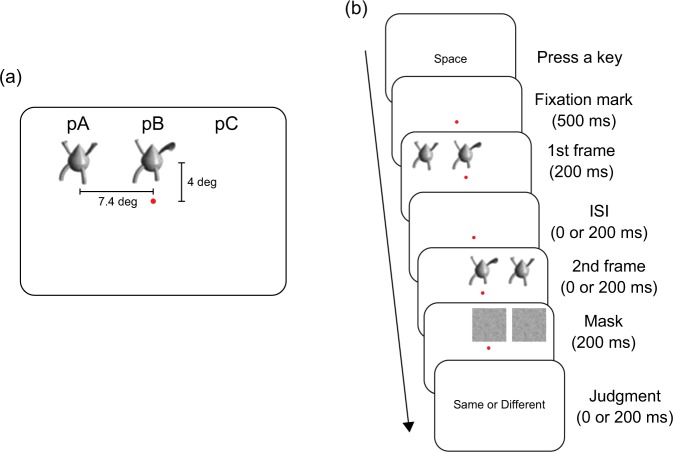
Examples of the position of the stimuli (**a**: the stimuli are above the fixation mark, and one of them is presented at pA) and the timeline of a trial in Experiment 1 (**b**: the target identity is same between the frames).

We presented the shape stimuli at the three locations (pA, pB, and pC; Fig. 2a). pB was below or above the fixation mark. pA and pC were on the left and right sides of pB, respectively. Figure 3b shows the timeline of a trial. Two objects (Objects 1 and 2) were presented for 200 ms (first frame). Then, Objects 1 and 2 reappeared and lasted for 200 ms (second frame). After the second frame, two squares of white noise were presented for 200 ms. In the first frame, we presented one of the two objects at pB and the other at pA (or pC). In the second frame, we presented one of the two objects at pB and the other at pC (or pA). The object identity at pB was identical between the frames in half of the trials but different in the rest of the trials. In the former case, the object identity at pA and pC was also identical between the frames but different in the latter case. The participants were asked to judge whether the object in the centre (i.e., the objects in pB) was identical between the first and second frames. Two kinds of ISIs were used (0 and 200 ms). We calculated *d prime* (*d*’) and *criterion* (*c*) based on the signal detection theory. As in the previous studies^10–12^, we used *c* as the spatial congruency bias index. Negative and positive values of *c* indicate the same and different bias, respectively.

**Figure 4:**
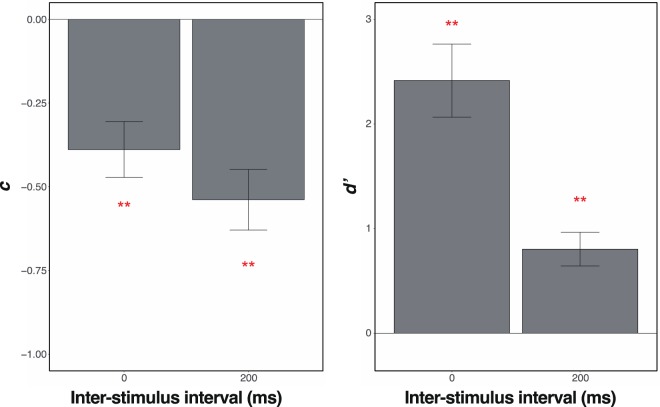
The results of Experiment 1. The vertical lines indicate *c* (**a**) and *d’* (**b**). The horizontal lines indicate the ISI. The error bars show the standard errors of the mean.

The results are shown in Fig. 4a,b. The *c*s were significantly lower than zero in both of the ISI conditions (0 ms, *t*(15) = 4.66, *p* < 0.001, Cohen’s *dz* = 1.16; 200 ms, *t*(15) = 5.95, *p* < 0.001, Cohen’s *dz* = 1.49). The *d*’s were higher than zero in both of the ISI conditions (0 ms, *t*(15) = 6.92, *p* < 0.001, Cohen’s *dz* = 1.73; 200 ms, *t*(15) = 4.98, *p* < 0.001, Cohen’s *dz* = 1.25). The results have revealed that the spatial congruency bias occurred not only in the 0-ms but also in the 200-ms ISI conditions even though the object’s identity was discriminable to some extent. Therefore, the spatial congruency bias was based on retinotopic processing, being tolerant of perceived motion (see also below for the preliminary experiment on perceived motion with the current stimulus configuration).

### Experiment 2: Judgment of the identity of the central and peripheral objects

**Figure 5:**
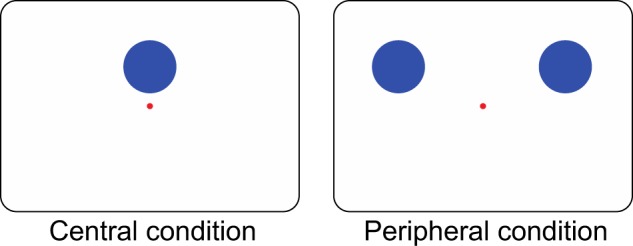
Examples of the indicators in Experiment 2.

Experiment 1 showed that the spatial congruency bias occurred irrespective of the ISI. However, it is premature to conclude that the spatial congruency bias was elicited indeed by retinotopic processing, as only the central location was critical (or task-relevant) for the participants in Experiment 1 to perform the judgment task. In other words, the task required participants to preferentially use the retinotopic locational information (i.e., the central location) in judging objects, which might boost the spatial congruency bias for both the ISIs. To address this possibility in Experiment 2, we asked participants to judge whether the object identity on both sides (periphery condition), as well as in the centre (central condition), was identical, which therefore made the central location non-preferential (Fig. 5).

**Figure 6:**
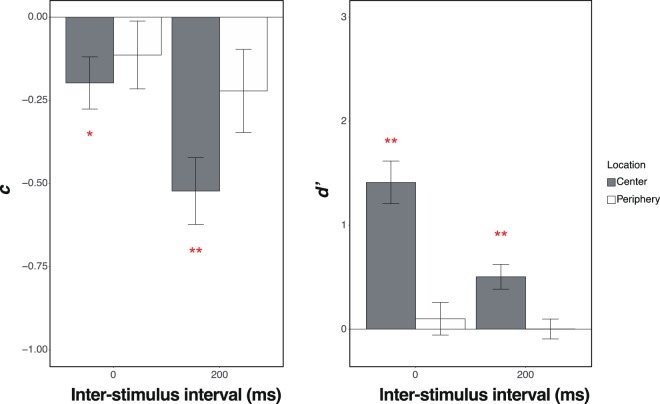
The results of Experiment 2. The vertical lines indicate *c* (**a**) and *d’* (**b**). The horizontal lines indicate the ISI. The grey and white bars indicate the results of the central and periphery conditions, respectively. The error bars show the standard errors of the mean.

The results of Experiment 2 are shown in Fig. 6a,b. In the central condition, the *c*s in both of the ISI conditions were significantly lower than zero (0 ms, *t*(15) = 2.52, *p* = 0.02, Cohen’s *dz* = 0.63; 200 ms, *t*(15) = 5.20, *p* < 0.001, Cohen’s *d* = 1.30). However, in the peripheral condition, the *c*s in the both of the ISI conditions were not significantly different from zero (0 ms, *t*(15) = 1.12, *p* = 0.28, Cohen’s *dz* = 0.28; 200 ms, *t*(15) = 1.77, *p* = 0.10, Cohen’s *dz* = 0.44).

In the central condition, the *d*’s were higher than zero in both of the ISI conditions (0 ms, *t*(15) = 6.87, *p* < 0.001, Cohen’s *dz* = 1.72; 200 ms, *t*(15) = 4.21, *p* < 0.001, Cohen’s *dz* = 1.05). In contrast, in the peripheral condition, the *d*s in both of the ISI conditions were not significantly different from zero (0 ms, *t*(15) = 0.64, *p* = 0.53, Cohen’s *dz* = 0.16; 200 ms, *t*(15) = 0.02, *p* = 0.99, Cohen’s *dz* < 0.01).

Even though the central location was non-preferential, the spatial congruency bias reliably occurred in the central condition, while this bias was not found in the peripheral condition. Thus, the results suggest that retinotopic congruency between the frames is responsible for the spatial congruency bias.

## Discussion

The present study examined whether the spatial congruency bias involved retinotopic, rather than non-retinotopic processing using the Ternus-Pikler paradigm. In the results, the spatial congruency bias consistently occurred when the location of the targets was identical between the frames. These results are in favour with the findings of the previous study using the SSPP^12^, strongly suggesting that the spatial congruency bias is based on retinotopic processing, and non-retinotopic processing contributes little to this bias.

What does retinotopic spatial congruency bias indicate? The previous studies proposed that location serves as a pointer, index, or object file to assign various kinds of features to an object^8,9^. Thus, the visual system possibly utilises location information for the identification of objects. Considering these, when objects are sequentially presented at the same location, our visual system basically judges (or mis-judges in the current case) that the objects are identical based on the shared location index, resulting in the spatial congruency bias^10–12^. Advancing this knowledge, the present and previous^12^ findings provided additional evidence: Retinotopic location information serves as the location index. This is in contrast with previous findings showing that retinotopic representations and spatiotopic representations were involved with lower-^22–24^ and higher-level^22,25,26^ visual areas, respectively. However, a recent study has demonstrated that retinotopic representations are also involved with higher-level ventral stream areas engaging in object recognition (e.g., lateral occipital complex)^27^. Briefly, these higher-visual areas possibly process object identity information based on retinotopic location. This is consistent with our findings.

It is possible that the object in the 1st frame influenced the appearance of that in the 2nd frame because of visual masking^28^, which might lead to the spatial congruency bias. The previous studies presented irrelevant visual masks between the frames to eliminate forward and backward masking effects^10–12^, while the present study did not present the visual masks after the 1st frame because of the aim of manipulating apparent motion. The duration of the frames and maximum ISI were 200 ms in the present study. These masking effects usually take place shorter than 200 ms^28^. Thus, if visual masking fully explained our results, the spatial congruency bias would disappear in the 200-ms condition; the results do not show this. Thus, visual masking cannot fully explain the present results. However, masking effect might remain over 200 ms dependently on the task and thus direct examination for this issue might be necessary in future studies.

Previous studies conducted a practice phase to determine the morph distance of the stimuli, which were used for the identification task in the test phase^10–12^. According to the performance of the practice phase, they adopted the morph distance, where the accuracy was within 70–75%. They also excluded the participants whose performances were below 55%. We did not conduct this calibration. Thus, it is possible that an extreme difficulty in identifying the objects led to the spatial congruency bias in the present study. To confirm this, we posteriori calculated the accuracy in Experiment 1 and the central condition of Experiment 2. The results showed that the performance of only one participant in Experiment 1 was below 55%, and the spatial congruency bias still survived (*t*s(14) > 5.59, *p*s < 0.001, Cohen’s *dz*s > 1.44) even if we excluded this data. Moreover, the detection sensitivity was low in the periphery condition of Experiment 2, indicating that identifying the objects was difficult in this condition. If the difficulty in identifying the objects simply led to the spatial congruency bias, the bias would also occur in the periphery condition of Experiment 2; however, this was not the case. Taken together, we can therefore reject the possibility that the difficulty in identifying the objects mediated the present results.

It is arguable that the participants paid attention only to the central position in Experiment 2 and this mediated in the results. If the participants’ attention was split in Experiment 2, the *d*’s should be lower in the central condition of Experiment 2 than in Experiment 1. Thus, we computed te averaged *d*’s in both the ISI conditions for Experiment 1 and those in the central condition of Experiment 2, and then conducted a two-tailed *t*-test for them. The results showed that the *d*’s were significantly lower in the central condition of Experiment 2 than in Experiment 1 (*t*(30) = 2.42, *p* = 0.02, Cohen’s *d* = 0.86), indicating that the participants’ attention was split to perform the task of Experiment 2.

A further previous study has examined whether the spatial congruency bias is sensitive to spatio-temporal contiguity cues (i.e., movement)^11^. In this study, the target object appeared and started to move after a short duration. Then, the object disappeared shortly after it reached the end point. The participants were asked to judge whether the object identity was identicl between the start and end points. In this case, the spatial congruency bias occurred despite incongruence in the retinotopic locations between the start and end points under limited parameters (i.e., each stimulus duration was 250 ms). That is, the spatio-temporal contiguity cues functioned in this condition. That said, we think that such the non-retinotopic bias based on the spatio-temporal contiguity does not conflict with the current finding (the absence of the bias under the periphery element-motion condition of Experiment 2). It is noteworthy that participants in the previous study were able to predict the fate of the moving object, whereas those in our study were unable to make predictions because they perceived motion only after the onset of the second frame. The continuous motion might elicit bias by the predictive mechanism, which would be different from the mechanism underlying the current retinotopic spatial congruency bias.

Converging evidence therefore suggests that the spatial congruency bias is based on retinotopic processing and that it is not affected by the spatio-temporal contiguity cues^12^. This further indicates that object representations are established not only at the spatiotopic coordinates but also primarily at the retinotopic coordinates in our visual processing. Given that the retinotopic object representation has less utility and is thus ecologically invalid, there should be some sort of ecologically-favourable (or goal-directed) compensatory system that establishes stable spatiotopic representations from the otherwise low-level retinotopic representations. This idea is consistent with the discussion of previous research^12,29^.

In general, two consecutive objects of different features may be judged as either a changing single object (same identity but different appearances) or two individual objects (different identities). In this context, the present, as well as previous studies^10–12^, required participants to judge whether the central object is identical in all features (i.e., the appearance judgement task) regardless of how participants perceived object identity (i.e., the number of object identities). However, the present study has illuminated this issue, by independently manipulating object identity using apparent motion (0-ms vs. 200-ms conditions). As a result, when participants perceived the central static object as a single object due to element motion under the 0-ms condition, it tended to be judged as the same; this also occurred even when participants perceived the central objects as two individual objects due to group motion under the 200-ms condition. In other words, participants missed detecting the appearance difference not only for a single object (i.e., same identity) but also for two individual objects (i.e., different identities). This indicates that the location-based identification robustly occurs for two separate objects independent of spatio-temporal contiguity, suggesting that the visual system might be overdependent on location information when identifying the objects.

## Methods

### Experiment 1

#### Participants

Sixteen volunteers participated in the experiment (seven males and nine females, mean age ± S.E.M. = 20.81 ± 0.47). This sample size was based on the previous study^10^, whose effect size (Cohen’s *d*) and statistical power (1 - β) were 1.01 and 0.96, respectively. All participants were unaware of the aim of this experiment and reported that they had normal visual functions. The study protocol was approved by the ethics committees of Waseda University and the experiment was conducted according to the guidelines stipulated in the Declaration of Helsinki. We obtained written informed consent from all participants prior to the experiments.

#### Apparatus

The stimuli were presented on a 22-inch monitor. The resolution was 1980 × 1080 pixels, and the refresh rate was 100 Hz. The presentation of the stimuli and data were controlled by a computer. The stimuli were generated by MATLAB (The MathWorks, Inc., Natick, MA, USA) with the Psychtoolbox extension^30,31^.

#### Stimuli and procedure

The viewing distance was 57 cm. Stimuli consisted of a fixation mark, shape images, and white background. The fixation mark was a red circle (radius = 0.2 degrees) and presented at the centre of the display. In previous studies of the spatial congruency bias^10–12^, a set of novel objects was used, which was modified from the Tarr stimuli set (stimulus images courtesy of Michael J. Tarr, Center for the Neural Basis of Cognition and Department of Psychology, Carnegie Mellon University; www.tarrlab.org). In the series of Golomb’s studies^10–12^, researchers selected 10 pairs of objects and morphed the images. One of these previous studies^10^ clearly stated that ‘each of these 10 families contained 20 individual exemplar objects (5% morph difference between each image)’ and the other studies^11,12^ conformed this. However, when two images are morphed, and the morph difference between the images is 5%, the number of individual images should be 21. Thus, we assumed that they eliminated the images at one of the end points, and hence they used 20 individual exemplar objects for each family. We selected the images at 5%, 50%, 55%, and 100% morph levels from each family, and thus we used 40 images as the stimuli. The size of each image was 5.9 × 5.5 degrees. We presented the stimuli at the three locations (pA, pB, and pC; Fig. 3a). pB was below or above the fixation mark, and the distance between the centre of the fixation mark and that of the image at the pB was 4 degrees. The pA and pC were from the left and right side of the pB, respectively, and the distance between the centre of the image at the pB and that of the image at the pA or pC was 7.4 degrees.

The experiment was conducted in a darkened room. The participants sat in front of the display, and their head position was stabilised using a head and chin rest. Figure 3b shows the timeli ne of a trial. The participants initiated each trial by pressing a spacebar. Then, the fixation mark was presented, and this persisted throughout the trial. Before the experiment, we told participants to focus on the fixation mark throughout the trial. After 500 ms passed, two objects (Objects 1 and 2) were presented for 200 ms (first frame). Then, Objects 1 and 2 reappeared and lasted for 200 ms (second frame). After the second frame, the two squares of white noise were presented for 200 ms. In the first frame, we presented one of the two objects at pB and the other at pA (or pC). In the second frame, we presented one of the two objects at pB and the other at pC (or pA). The vertical position (i.e., above or below the fixation mark) and initial peripheral position (i.e., pA or pC) were determined randomly across the trials. The participants were asked to judge whether the object in the centre (i.e., the objects in pB, which we call the targets) was identical in all features between the first and second frames. If two consecutive objects have different features, they may be judged as a changing single object (same identity but different appearances) or two individual objects (different identities). Thus, this study required participants to detect (subtle) differences in appearance, following previous research^10–12^. The combination of Objects 1 and 2 was 5% *vs* 50% or 55 *vs* 100% from the same family. Two kinds of ISIs existed between the first and second frames (0 and 200 ms). Moreover, two kinds of combinations of the two objects and positions were manipulated as a factor of target identity (same or different). Under a same condition, Object 1 consistently appeared at pB as the target between the frames, while the position of the Object 2 changed between the frames (i.e., pA → pC or pC → pA). In this case, the targets’ identities were the same between the frames. Under a different condition, both objects shifted horizontally (e.g., Object 1: pA → pB, Object 2: pB → pC); the targets’ identities were different between the frames (Object 2 in the first frame but Object 1 in the second frame). There were two ISI conditions (0 and 200 ms), two target identity conditions (same and different), 20 pairs of objects, and two repetitions: each participant performed 160 trials. The trial order was randomised across the participants.

Note that we preliminarily examined whether the perceived motion would depend on the length of the ISI with the current experimental parameters. In this preliminary experiment, 16 participants were asked to report which element or group motion they perceived. We calculated the proportion of ‘group motion’ responses for each duration (0 ms, *M* = 0.28; 200 ms, *M* = 0.84). To confirm whether the group-motion proportion was significantly different from a chance level (i.e., 0.5), we performed one-sample *t*-tests. The results showed that the proportion was significantly lower in the 0-ms condition than the chance level (*t*(15) = 3.38, *p* = 0.004, Cohen’s *dz* = 0.85), while the proportion was significantly higher in the 0-ms condition than the chance level (*t*(15) = 5.43, *p* < 0.001, Cohen’s *dz* = 1.36). These results are important evidence that element motion is predominantly perceived in the 0-ms condition, while perception of group motion is predominant in 200-ms condition. Thus, we confirmed that our experimental parameters were valid for assessing our prediction.

#### Data analysis

We calculated *d*’ and *c* based on the signal detection theory. As in the previous studies^10–12^, we used *c* as the spatial congruency bias index. Negative and positive values of *c* indicate the same and different bias, respectively. Our interest was whether the spatial congruency bias occurred, thus, we conducted one-sample *t*-tests comparing the *c* in each ISI condition to 0. Moreover, we were interested in the detection sensitivity, hence, we also conducted one-sample *t*-tests comparing the *d’* in each ISI condition to 0. The alpha level was 0.05, and we reported Cohen’s *dz* as the effect sizes.

Using *c*’ (=*c*/*d*’) might be desirable. However, using *c* is more beneficial for easily comparing the results across studies^10–12^. Moreover, when the *d*’ is zero, calculating *c*’ is impossible (incidentally, some *d*’s in the present study were zero). Therefore, the present study used *c*. For those who would like to calculate the *c*’, we could provide all the raw data (please see Data availability).

### Experiment 2

#### Participants, apparatus, stimuli, procedure, and data analysis

Sixteen volunteers participated in the experiment (11 males and 5 females, mean age ± S.E.M. = 20.93 ± 0.44). The method was identical to that of Experiment 1 except that the three possible object locations that participants were to pay attention to were all task-relevant. After the masks, we presented blue circles (radius = 3.35 degrees) for 500 ms as indicators to inform the participants about to-judge objects. In the central judgment condition, the circle was presented at pB, while the two circles were presented at pA and pC in the peripheral judgment condition (Fig. 5); two conditions were manipulated within block. We asked the participants to judge whether the objects indicated by the blue circles were identical between the frames. There were two ISI conditions (0 and 200 ms), two target identity conditions (same and different), and two kinds of the indicator position (centre or periphery), 20 pairs of objects, and two repetitions: each participant performed 320 trials. We calculated a *d*’ and *c* based on the signal detection theory. The alpha level was 0.05, and we reported Cohen’s *dz* as the effect sizes.

## Data Availability

All the data have been uploaded at https://figshare.com/s/c191acedbe75858b2d08.
